# The nearly complete mitochondrial genome of Chinese pygmy dormouse *Typhlomys cinereus* (Rodentia: Platacanthomyidae)

**DOI:** 10.1080/23802359.2016.1209092

**Published:** 2016-09-05

**Authors:** Xinfang Lv, Haiyan Cong, Lingming Kong, Masaharu Motokawa, Masashi Harada, Yi Wu, Yuchun Li

**Affiliations:** a Marine College, Shandong University, Weihai, China;; b The Kyoto University Museum, Kyoto, Japan;; c Laboratory Animal Center, Osaka City University, Osaka, Japan;; d College of Life Sciences, Guangzhou University, Guangzhou, China

**Keywords:** Mitochondrial genome, Platacanthomyidae, *Typhlomys cinereus*

## Abstract

In this study, we report the nearly complete mitochondrial genome of a Chinese pygmy dormouse *Typhlomys cinereus* (Rodentia: Platacanthomyidae). The 15,011 bp genome is consisted of 13 protein-coding genes, 16S rRNA, 21 tRNAs, partial 12S rRNA and control region. A phylogenetic tree was built using 12 protein-coding genes of 19 species from Dipodoidea and Muroidea. Our result shows that *T. cinereus* represents the earliest split within Muroidea. The genome would contribute to further study of phylogeny in Muroidea and Rodentia.

The Chinese pygmy dormouse *Typhlomys cinereus* belongs to the enigmatic family Platacanthomyidae which is currently composed of only two genera, *Typhlomys* and *Platacanthomys* (Musser & Carleton 2005). Before *T. c*. chapensis *was* elevated to species status by Abramov et al. (2014), researchers held the point that the monotypic *T. cinereus* was consisted of five subspecies according to external and skull morphologies (Wang et al. 1996; Musser & Carleton 2005). Up to now, there is only two molecular studies concerned with this species on the taxonomic position of subspecies *T. c*. *chapensis* and the phylogenetic position of Platacanthomyidae (Jansa et al. 2009; Abramov et al. 2014). Here we firstly provide and characterize the nearly complete mitochondrial genome of *T. cinereus* and explore the phylogenetic position by large scale of molecular data. The specimen of *T. cinereus* (G12230) used in this study for mitochondrial genome analysis was collected in Mt. Nanling, Guangdong province, China, and was stored in Marine College at Shandong University (Weihai).

As our result, the nearly complete mitochondrial genome of *T. cinereus* is 15,011 bp in length (GenBank accession no. KX397283). The genome was identified as containing 13 protein-coding genes, 21 tRNA genes, the 16S rRNA gene, and a partially sequenced 12S rRNA gene. However, the tRNA-Phe gene, partial control region and partial 12S rRNA gene are not sequenced in this study. The arrangement of these genes is same to other vertebrate. Among these genes, 27 are transcribed on the heavy-coding strand (H-strand), whereas the other 9 genes on the light-coding strand (L-strand). All the protein-coding genes start with ATN, except that ND1 adopts GTG as start codon. ND6 and Cyt*b* adopt AGG and AGA as stop codon respectively, the other protein-coding genes use TAN or incomplete T– as stop codon. The incomplete stop codons can be completed by post-transcriptional polyadenylation (Ojala et al. 1981). The identified 21 tRNA genes range from 66 to 75 bp in length. All the identified tRNA genes could be fold into canonical cloverleaf structure except for tRNA-Ser (AGY) in which its dihydrouridine (DHU) simply forms a loop.

The evolutionary relationship of Platacanthomyidae with Dipodoidea and Muroidea has been controversial for a long time (Alston 2009; Thomas 1896; Miller & Gidley 1918; Simpson 1945; Chaline et al. 2009). To explore the phylogenetic position of Platacanthomyidae, here we downloaded some available mitochondrial genome sequence of every representative subfamilies of Dipodoidea and Muroidea to build the phylogenetic tree. The maximum likelihood tree was constructed by the aligned 12 protein coding genes transcribed on the H-strand, the total length of the aligned sequence was 10,743bp. The phylogenetic result showed that *T. cinereus* is clustered with species of Muroidea and it was the earliest split within this clade (Figure 1). The result confirmed that of Jansa et al. (2009).

**Figure 1.**
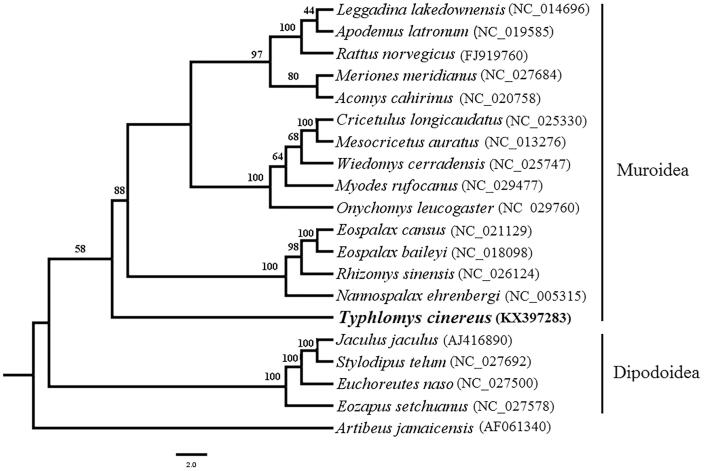
Maximum likelihood (ML) phylogenetic tree based on the 12 protein-coding genes under GTR + I + G model. ML bootstrap values are shown above nodes.

Unlike other rodents, species of family Platacanthomyidae is less studied, especially molecular studies. The nearly complete mitochondrial genome of *T. cinereus* reported in this study will provide more essential molecular data for further studies.
